# Adolescent girls' and parents' views on recruiting and retaining girls into an after-school dance intervention: implications for extra-curricular physical activity provision

**DOI:** 10.1186/1479-5868-8-91

**Published:** 2011-08-23

**Authors:** Russell Jago, Laura Davis, Jade McNeill, Simon J Sebire, Anne Haase, Jane Powell, Ashley R Cooper

**Affiliations:** 1 Centre for Exercise, Nutrition & Health Sciences, School for Policy Studies, University of Bristol, 8 Priory RD, Bristol, BS8 1TZ, UK; 2 Faculty of Health and Life Sciences, University of the West of England, Glenside Campus, Stapleton, Bristol, BS16 1DD, UK

## Abstract

**Background:**

Many adolescents are not sufficiently active and girls are less active than boys. Physical activity interventions delivered during curriculum time have reported weak effects. More sustained changes in physical activity may be obtained by facilitating participation in enjoyable activities. Dance is the favourite activity of UK girls but there is a shortage of dance provision. Dance sessions delivered after the school day could prove to be an effective means of engaging adolescent girls in physical activity. There is a lack of information about the factors that would affect girls' recruitment and retention in an after-school dance programme.

**Methods:**

Focus groups were conducted with 65, Year 7 (11-12 year old) girls from 4 secondary schools in Bristol. In-depth phone interviews were also conducted with 16 (4 per school) of the girls' parents. Interviews and focus groups examined issues that would affect recruitment into the intervention, strategies that could be used to attract girls who have little or no previous experience in dance, any factors that would increase their interest in participating in an after-school dance programme and any factors that would affect retention in the programme. All interviews and focus groups were digitally recorded and thematically analysed.

**Results:**

Girls reported that a taster session in which they had an opportunity to sample the intervention content and "word of mouth" campaigns by peers, who did not need to be their friends, would encourage them to participate in an after-school dance programme. Sessions that maximised enjoyment and facilitated socialisation opportunities would enhance retention. Parents reported that encouraging groups of friends to join the programme, and stressing the enjoyment of the session would increase participation.

**Conclusions:**

Recruitment and retention campaigns that focus on enjoyment, socialisation, mastery, goal setting and relating to other girls may be effective strategies for recruiting and retaining girls in an after-school dance programme. These factors are consistent with well-established theories of individual behaviour change such as self-determination theory and social cognitive theory. Recruitment and retention campaigns that are targeted to address theoretically derived mediators of behaviour may be more effective than traditional approaches.

## Background

Physical activity during childhood has been associated with lower levels of several health risk factors including insulin, glucose, blood pressure and body composition [1,2]. Physical activity is also associated with improved emotional well-being and self-esteem among young people [3]. Despite the benefits of regular physical activity, many young people do not meet the current UK recommendation of an hour of physical activity on most days of the week [4]. For example, data from the Bristol based Avon Longitudinal Study of Parents and Children (ALSPAC) indicated that only 5% of boys and < 1% of girls engaged in an hour of moderate to vigorous intensity physical activity per day [4]. Physical activity levels decline during childhood and the start of secondary school is a key period of change [5]. Girls are less active than boys throughout childhood and the age-related decline in physical activity is steeper for girls than for boys [5]. Thus, there is an urgent need to increase adolescent girls' physical activity.

Systematic reviews of youth physical activity interventions or obesity prevention interventions with a strong focus on physical activity have reported weak or no effects [6-8]. The majority of these interventions have been delivered at schools by changing existing curriculum provision [6]. Schools are, however, under considerable pressure to raise standardised test scores and as such the curriculum time that can be devoted to physical activity is increasingly limited. Physical activity sessions that are delivered through extra-curricular provision (i.e. before or after school) hold promise as a means of reaching large numbers of youth in a period that is less pressured by competing academic demands [9]. Novel physical activity programmes aimed at adolescent girls could be delivered during extra-curricular periods [9].

The successful delivery and evaluation of an extra-curricular physical activity intervention will require at least two critical elements: 1) that a sample representative of the target group is recruited for both the intervention and control arms (recruitment); and 2) that the sessions are sufficiently appealing to the participants in order to maintain their participation in the intervention (retention). These two issues are discussed below.

Recruitment is central to the success of research projects. Recruitment is often affected by the characteristics of the individual as well as broader social factors. Among adults, it is well documented that programmes aimed at helping people improve health behaviours tend to be taken up by middle class, well-educated individuals and those with pre-existing positive attitudes to their health [10,11]. Similar recruitment challenges have been reported among studies that have involved children and adolescents. For example, in the "Girlstars" programme, a physical activity intervention for 9 to 13 year old girls living in community housing projects in Boston, only 60 of the target of 80 girls (75%) were recruited [12]. The authors reported that recruitment was the biggest challenge in the project with the recruitment issues preventing a full evaluation of the intervention [12]. In the Trial of Activity in Adolescent Girls (TAAG) a US multi-centre trial, site-specific recruitment efforts were implemented, along with baseline recruitment efforts being adapted and increased in intensity for re-recruitment at the follow-up assessment [13]. For example, one of the intervention sites increased recruitment from 74% at baseline to 88% at follow-up after paying a teacher a small stipend to serve as the key recruitment contact; conducting small group presentations to potential participants instead of the usual large assemblies; and providing a $50 incentive for completing all measures [13]. While these flexible approaches are a testament to the research teams' ingenuity, they indicate the challenge of recruitment into interventions. Collectively, these studies highlight that the development and piloting of recruitment strategies would ideally form a central role in the design of complex interventions [14].

Previous research has indicated that parental support for physical activity is a key predictor of children's physical activity with this influence enduring into adolescence [15,16]. Parental support is also a critical factor in recruiting and retaining youth in physical activity interventions. For example, in a pilot study of a school-based nutrition and physical activity intervention, Slawta and colleagues recruited an intervention group of 45 students and a much lower number of 20 control group participants [17]. The authors reported that parents of children in the control school did not feel it was appropriate for their child to participate in assessments when they were not going to benefit from participating in the intervention [17]. Similarly, US researchers have noted that many parents are unwilling to allow their children to attend extra-curricular programmes because attendance would prevent the use of the school bus network for transport home [18]. These examples highlight the importance of parental influence on child behaviour and a need to examine the ways parents can support participation in an extra-curricular programme.

Dance is a unique form of physical activity that may be sufficiently appealing to adolescent girls to encourage participation across the lifespan. Dance is the favourite form of physical activity among UK secondary school age girls [19] and is recognised by several peer groups as being a desirable activity in which to engage. Girls who would normally drop out of most other forms of physical activity during secondary school will engage in dance if it is available to them [20]. Thus, an extra-curricular dance programme holds promise as a unique means of engaging adolescent girls in physical activity. There is, however, a shortage of information about recruitment and retention of adolescent girls into this type of dance intervention and its clash with other demands on leisure time after school. There is some evidence for the preferences of adults regarding the use of leisure time [21], but minimal evidence for the way preferences for using leisure time play into decisions to attend dance classes or other leisure activities in adolescent girls.

In light of the evidence presented above, the aim of this paper was to identify the key factors that would affect recruitment and retention into an after-school dance programme with a focus on both child and parent perspectives. We also specifically sought to identify information that could be translated to other extra-curricular physical activity interventions and potential links to behavioural theory on which future interventions could be based.

## Methods

### Participants

The participants were Year 7 (11-12 year old) girls and a sub-sample of their parents. A total of 65, Year 7 girls were recruited from four co-educational secondary schools in Bristol, UK. As schools within the greater Bristol area fall under the jurisdiction of two school districts, we recruited two schools per district. Schools were recruited based on a measure of income deprivation [22] derived from the income characteristics of the area of residence for each child on the school roll. The percentage of families with children receiving Child Tax Credits and/or Working Tax Credits informs the deprivation indicator. Based on a median split of deprivation scores, we recruited one high and one low deprivation school from each district.

A recruitment session was held for all Year 7 girls in each school, where all girls were invited to participate in a research study about physical activity and dance. Potential participants were provided with an information sheet about the project and a consent form. Children were provided with an information sheet and a letter for their parents inviting them to participate in a phone interview to discuss similar issues.

All Year 7 girls in each school were invited to participate in the focus group. The recruitment rate varied between 23% and 27% at the four schools with all students who consented and were present at the school on data collection days (3 students were absent on data collection days), taking part in the study. Sixteen parents (two fathers), of Year 7 girls (4 parents from each of the four schools) were randomly sampled from the 47 parents who volunteered to participate. The sample was limited to 16 parents as theoretical saturation, where no new information was obtained, was deemed to have been achieved. The study was approved by the School of Applied Community and Health Studies Ethics committee at the University of Bristol and informed parental consent (for girls' participation) and informed consent (for parental participation) were obtained for all participants.

### Girls' data collection

Focus groups were chosen as a method of data collection for the girls. Focus groups facilitate development of thoughts and ideas through participant interaction in a comfortable, safe and supportive environment [23,24]. Two focus groups were held at each school, with an average of 8 participants in each focus group. Each focus group lasted 35-50 minutes and was digitally recorded using an Olympus DS-22 recorder.

The focus groups had a semi-structured design with follow-up probes on key topics of interest. Questions were developed and piloted in one school before being finalised.

The focus group questions explored factors that would influence recruitment into the intervention. The participants were asked to identify factors that would increase their interest in taking part in an after-school dance programme (such as the style of dance, the type of music etc) and also what strategies could be used to attract girls who have little or no previous experience in dance. The focus groups were facilitated by LD and JM.

### Parental data collection

In previous research, we have found it difficult to schedule a time when multiple parents can attend a focus group [25]. To address this issue we conducted in-depth, semi-structured phone interviews with parents. Phone interviews encourage interviewees to answer questions of a delicate nature as the interviewer is not present [26,27]. Interviews were conducted by experienced interviewers (LD and JM) and were based on two pilot interviews. The mean interview length was 15 minutes. The parental questions were designed to assess comparable constructs to the girls' focus groups and focused on issues that might have prevented the girls from attending; parents willingness to pay for their daughters to attend and associated price limits; strategies to assist the recruitment and retention of girls who did not currently dance; and any ways that the parents could support their daughters participation in the programme.

### Analysis

All focus group and interview recordings were transcribed verbatim and anonymised. As the data are considered exploratory, we adopted a thematic analytical approach [28] with the same three stage analytical process being applied independently to the focus groups and interviews. First, key themes were identified by reading the transcripts line by line (LD parent/JM child) and marking the text with codes that described the content of the response [29]. Codes were entered as free nodes into newly created databases in NVivo (Version 8, QSR, Southport, UK). Second, an additional team member (LD child/JM parent) reviewed all transcripts, resulting codes and differences in coder interpretation identified. (A third team member (SJS or RJ) acted as the arbitrator of actual differences in interpretation). Third, hierarchies of codes were created and summarised and themes within each group were developed. Potential quotes that were deemed to best represent the nature of each theme were then extracted, discussed by the authors and a final selection of quotes produced.

## Results

The sections below provide a summary of the information provided by the girls and their parents.

### Girls focus groups

#### 1) Barriers to overcome during recruitment

The girls reported three main factors would affect their recruitment into the intervention: 1) perception of the intervention as fun and enjoyable; 2) if the intervention provided opportunities for socialising with other girls; and 3) if the activity sessions did not clash with existing commitments. Support for each of these themes is presented below.

### Perception of the intervention as fun and enjoyable

The majority of the girls reported that they would attend a dance programme if it was perceived to be fun and enjoyable. The girls also reported that dance was perceived as an opportunity to get together, develop new skills and spend time doing something that they enjoyed with others who have a similar interest.

*'Sometimes I like dance in my room all the time and it will be fun if we got into the groove and did it'*(Child 36, School 2).

*'It's a chance for people to get better at things they can't do' *(Child 29, School 3).

*'Because I already prance around in my front room dancing and singing and I like dancing. It's one of my passions. I want to meet other people that like dancing as well as I do' *(Child 19, School 2).

The facilitator attempted to probe to draw more information about how make the sessions enjoyable but the students were not able to articulate responses on how to address this issue.

### Socialising opportunities

The girls reported that dance would provide a good opportunity to socialise and make friends without the usual associated school pressures. Furthermore, most girls reported that they would still attend the dance programme even if their friends didn't because they were open to the idea of making new friends.

'If your friends go there it's a chance to hang-out, like without school pressure' (Child 29, School 2)

*'Because it would give me a chance to mix with other people who like doing the stuff that I do' *(Child 19, School 2).

*'Because if none of my friends went, it's nice to meet different people' *(Child 51, School 4).

*'So instead of being out on the streets or whatever, playing with your mates or whatever, instead of being bored just sat in a park or something you could be there after school and then you could like walk home with them' *(Child 21, School 2).

### Clash with other opportunities

The major factor that was identified as a potential reason for non-attendance at an extra-curricular dance programme was conflict with other after school activities, both physical and non-physical, that the girls might wish to attend.

*"I might have a club that day, I have to go to, because I do a lot of things after school and I might not be able to come because I've got too much on or what ever" *(Child 28, School 2).

*"It might clash with different things that you originally do" *(Child 41, School 3).

*'Giving up other clubs that you have to make commitment to because you had like a big thing coming up.' *(Child 44, School 3)

#### 2) Strategies to increase recruitment

The girls considered that an opportunity to sample the nature of the dance classes in a taster session would enable them to fully understand what they were committing to and would likely increase recruitment. Another viable approach to recruitment involved word of mouth campaigns from peers encouraging them to participate in the intervention. Each of these themes is discussed below.

### Taster session

The majority of participants wanted an opportunity to experience the intervention before committing to attendance. The girls reported that a "taster session" would provide them with an opportunity to obtain an appreciation of what the intervention would involve, without the pressure of signing up.

*'Maybe like a trial that says one week so that everyone can go and then if they don't like it, they don't have to go again but if they do they can carry on coming' *(Child 44, School 3).

*'So if you're in a lesson, for like five minutes, take a group time out of the lesson and then just say get them to do dance in a hall or something and show how fun it can be. And then because they'll come after school and see if they like it.'* (Child 28, School 2).

### Word of mouth campaigns & peer support

The girls reported that they would be encouraged to attend a dance programme if their friends encouraged them to do so. The girls also suggested that positive encouragement from other individuals within the group, who were not necessarily their friends, would provide an incentive for them to take part in the programme.

*'Say we started the club because all of us here really enjoyed it and then we'd go to our friends and then they'd join in and they'd go tell their friends and they'll join. So in the end we'll have loads of groups that get along and enjoy dancing after school' *(Child 18, School 2).

*'Say if like one group went dancing for a time and they thought it was really good we could like say to them like we thought it was really good, you should join or something to give them like news about it and they might think about it and join' *(Child 40, School 3).

*'If we say it is going to be fun and if they feel embarrassed they can stay with us, we could help them out until they get used to it' *(Child 8, School 1).

*'We could be friendly and welcome them to the group, help them if they are stuck.' *(Child 54, School 4).

#### 3) Sustaining participation

Four factors emerged that would influence whether the girls sustained participation in the intervention: 1) the type of dance; 2) the type of music; and 3); if the girls were provided with an opportunity to gain some input over the nature of the sessions.

### Dance genre

The girls indicated that the type of dance would influence their recruitment into the programme with a clear preference for modern types of dance, such as hip hop. They favoured this style of dance due to its high energy beats and its changing dynamics which they perceived as making the dance more interesting and fun.

*'I kind of like street dance' *(Child 37, School 3).

*'I would think that most people enjoy hip hop [okay]. I don't know why but my opinion would be hip hop' *(Child 10, School 1)

*'Hip hop and street because when you've got all the moves it just looks totally amazing and if people see something that's totally amazing, then they'll want to do it and learn more' *(Child 19, School 2).

*'I like street dance because like when you like watch a film and they're all doing this street dance I really want to learn how to like do the moves and stuff'* (Child 46, School 3).

### Using upbeat current music

Most of the participants stated that they wanted to dance to current chart music as this was something that they could relate to whilst keeping them motivated.

*'Yeah its sort of like quite jumpy and stuff' *(Child 44, School 3).

*'Because you're always quite moving, you're not always really slow and when you like watch it, say if it's on video, if you watch it back you think oh yeah that looks really good' *(Child 46, School 3).

'*Yeah, I like current stuff or I like older music, not like really old but sort of... not in the charts but recently been in the charts' *(Child 42, School 3)

'*Like loud stuff. Not like too loud but like energetic stuff' *(Child 46, School 3).

### Pupil input into the session content

The girls believed it was important to have the opportunity to provide some input into the dance sessions, thereby facilitating a sense of ownership of the sessions. In addition, girls also believed that being able to set a goal and work towards it would keep them motivated to continue their participation in the programme.

*'It feels like you have actually worked it [i.e. the girls created the dance], not somebody else's that they have made' *(Child 64, School 4).

*"Because you can do like ... you can like learn a dance and then halfway through you can put your own moves in and that would be good because like it doesn't have to be all choreographed [yeah] by your dance teacher, so you could put your own moves in to show people how well you can dance' *(Child 25, School 2).

*'Or like maybe not always the teacher's moves. Like "A" said like we can put some of our own moves in so if we couldn't do that move because not everyone can do it we can all like say like well we would like to be in the actual dance like something that we could actually do' *(Child 40, School 3)

*'I don't know it just sort of gives you a bit more freedom, not just being told what to do' *(Child 57, School 4*)*

*'Because it feels like ... it's actually what you've worked at and its not somebody else's that they've made and then we had to copy them. You've actually worked yourself and used your creative thing that you actually made up some dance yourself' *(Child 58, School 4)

### Parent interviews

#### 1) Parental suggestions of ways to increase participation

Parents reported three main factors that would affect their daughter's' participation in the intervention and could be used to increase recruitment: 1) attracting groups of friends into the intervention; 2) stressing the health and fitness benefits of participation; and 3) using a taster session to allow the girls to sample the content of the sessions.

### Attracting friends

Parents strongly suggested that targeting attendance by groups of friends would potentially increase the number of girls recruited into the programme. They explained that girls may find it daunting and intimidating to sign up for the dance programme alone. It is important to note that this was different to the girls' perspective that they would attend with or without their friends.

*'I think to get some of their friends, obviously their close friends if they're involved in it as well to maybe talk with their friends [right] and get their friends on side and maybe this would persuade their children [right] to take part' *(Parent 1)

*'I think if there's a group of them, if they're in a group and that they know each other, I think that's quite a key issue to sort of get them along in the first instance as opposed to going there on their own and not knowing anybody'* (Parent 2).

### Health and fitness

Parents thought that stressing the health and fitness benefits of dance to the girls would help during both the recruitment phase and throughout the intervention. It is thought that if the girls feel that the programme is beneficial to their health and fitness rather than having a competitive focus, they will be more likely enrol in, and persist with the programme.

*'Like I say especially if it was somebody that thought that they, that might be one of the off-putting things for them that it was going to be too competitive [right] maybe a dance class that's not particularly geared for ... for competition as such but they, as a, as a more general idea as to, as a keep fit approach [right] rather than a competitive approach might encourage more people to actually do it" *(Parent 4)

*'I think it's a very good activity. It can cover all age groups, not just Year 7. It's a physical activity in this day and age where the poor kids are getting it on their backs all the time from they've got to do this amount of exercise, they've got to have five fruit and veg a day. It doesn't matter whether you come from a rich family or a poor family, it's a nice get together thing' *(Parent 4)

### Taster session

Parents thought that the biggest barrier to recruitment would initially be getting the participants to sign up for the programme. Like the girls, the parents thought that a taster session would be a useful means of allowing the girls to sample the type of intervention before deciding whether to participate. The parents also suggested that if the session was delivered during a PE lesson it would be possible to target all of the girls at once. Delivering the taster session to all girls during PE would also limit the possibility of only girls who currently participate in dance from attending.

*'Perhaps do a taster session or something during a, I don't know if you do it during a school P.E. lesson so that everyone can see that it can be everyone that can be involved' *(Parent 3)

*'I would but not just a one-off taster session because it might not be the dance that they like it might be, if you did two or three with different dance ... yeah, dance styles [yeah] that might pull them in a bit more' *(Parent 5)

#### 2) RETENTION/SUSTAINING GIRLS INTO THE PROGRAMME

Parents reported that three factors would affect whether the girls sustained their participation in the intervention once it had started. These three factors were: 1) whether the girls set goals to increase their commitment during the intervention; 2) whether the sessions were enjoyable; and 3) the characteristics of the teacher. These themes are discussed below.

### Goal Setting

All parents noted the importance of goal setting to help retention rates during the ten week programme. The parents suggested that setting achievable goals and targets from the start of the dance programme would give the girls something to work towards, both as individuals and as a group.

*'Well again I suppose making sure that it was, that they were giving something that was achievable for them [right] so I suppose that sense of achievement however ... small it might be is rewarding to them.' *(Parent 4)

*'... yes, if you give a child a goal obviously, like I say, some pick it up quicker than others, they will work the best they can, do the best they can, towards that' (*Parent 4*)*

### Enjoyment

Parents stressed the importance of the dance session being enjoyable and fun to help retention rates throughout the ten weeks.

*'If they can see what it entails, how much fun it can be, not that it's exhausting or anything like that but just if they could probably meet friends, a chance to chill out and enjoy themselves. Do something that's interesting I think that would entice them to go.' *(Parent 6)

*'Yeah, yeah really stress that just everyone's welcome enjoy, and it's, yeah as you say really informal that's [...]Really informal, all you need is a pair of trainers because most kids have got trainers, just turn up with your trainers and come have a, an hour of fun' *(Parent 3).

### Importance of the dance teacher

Parents thought that the dance teacher and his or her delivery/interaction style would affect both the recruitment and retention rates for the study. The parents stressed the importance of selecting someone who is reliable, has an understanding of the needs of young people (year 7 girls) and of issues that may arise within the session that may or may not be related to dance.

'Yeah because obviously she's got to engage all the children and if she finds that there are children that are thinking of wanting to drop out then obviously she needs to get to the bottom of it and find out why and if it is, maybe they're being bullied by the other children in the class and they don't want to say obviously to their parent or to their teacher but yeah, she needs to be aware of all the children, what's going on and [yeah] make sure that doesn't happen.' (Parent 3)

*'You've got to make sure it's the right person teaching the children [okay] and also the right music. You don't want someone doing a dance class they don't know anything about music because obviously the kids know more nowadays than we do about music so I think it's very important that whoever's teaching them has got the right music on and stuff so. So that's going to be really important whoever's teaching' *(Parent 4).

## Discussion

The findings presented in this paper highlight a number of strategies that could be used to increase recruitment into an extra-curricular dance programme and then retain the girls once they have joined the study. A number of the key themes proposed by the parents and children to increase recruitment and retention are also consistent with psychological theories of behaviour change. Two frameworks with which our findings are particularly consistent are self-determination theory (SDT) [30] and social cognitive theory (SCT) [30,31] suggesting that addressing key components of these theories when planning how to attract participants might enhance recruitment and retention rates. As such, in the sections below we have highlighted each of the key themes, implications for recruitment and retention and links with both SDT and SCT.

Parents suggested that targeting groups of friends would facilitate the recruitment of a diverse group of girls who do not currently dance, while the girls suggested that they would attend without their friends if they thought the activities were enjoyable. We have previously shown that 10-11 year old girls who are active with their best friend outside of school, engage in a greater intensity and volume of physical activity than those who are only active with their friends at school [32]. Results are consistent with the relatedness principle of SDT which suggests that the degree to which people feel understood by and connected to significant others around them underpins adaptive forms of motivation to engage in a behaviour [30]. The ASSIST study reported that a peer-led intervention helped to reduce the prevalence of smoking among secondary school students [33]. Rather than utilising the best behaved or most studious pupils as the peer leaders, ASSIST used the opinion makers as the peer leaders. When the ASSIST findings are combined with the data presented here and situated within SDT it is possible to argue that using peer leaders to create a "buzz" about a new activity may be a useful means of increasing recruitment into after-school physical activity interventions. Moreover, the success of these campaigns may be further enhanced if groups of friends who do not currently take part in the activity enrol together, thereby providing mutual support (i.e. relatedness) for participation.

Both the girls and their parents were clear that they wanted to sample the activities before deciding whether to participate. This suggestion is ethically sound, as it allows the girls to make a fully informed decision about whether to join the study [34]. The adoption of a taster session will also encourage researchers to check that the new activity will engage the participants. It is therefore essential that the taster session reflects the intervention and is presented by the staff member that would ultimately be responsible for delivering the intervention.

Enjoyment [35-37] and socialisation opportunities [36,38] are strong predictors of girls' physical activity and the participants in this study indicated that both perceived enjoyment and socialising opportunities would positively influence their decisions to take part in an after-school activity programme. These concepts are consistent with the intrinsic motivation and relatedness principles that are central to SDT [30] and suggest that designing interventions that provide opportunities for enjoyment and socialisation is likely help retain girls in an extracurricular intervention. Stressing the enjoyment and socialisation opportunities during recruitment may also help to attract girls that would not normally be attracted to the intervention.

Parents and children suggested that ensuring that the girls had a sense of ownership over the activity would increase retention. This concept is consistent with the autonomy-support, competence, autonomy and relatedness needs, and autonomous motivation principles of SDT [30]. Consistent with the self-efficacy construct that underpins SCT the girls also suggested that setting achievable goals that build over the course of the programme would increase retention. Interpreting these comments in relation to psychological theories of behaviour change suggests that recruitment and retention campaigns that are targeted to address key mediators of the outcome behaviour may be effective. Although theoretical concepts have been examined as potential mediators of behaviour change [37,39] we are not aware of any study in which these factors have been examined as potential predictors of study recruitment or retention. As such we believe that focussing on the mediators of recruitment and retention in behavioural interventions may be provide a new opportunity to understand how researchers can motivate participants to engage in behaviour change programmes.

Music and type of dance were two dance specific issues that were highlighted by the girls as factors that might affect recruitment. The girls were very clear that up-tempo and current music would positively affect their willingness to attend the sessions. This is consistent with data that has reported that high tempo music was positively associated with intrinsic motivation and participation in traditional Greek dance [40]. Furthermore, the girls also believed they would be more likely to join the study if more modern/urban forms of dance were delivered. It was noticeable that the girls did not seem to have a clear understanding of what the forms of dance were, but as noted above more modern forms of dance are currently popular within the UK. It may therefore be the case that delivery of alternative forms of dance may also be appealing to the girls if they understand what they are and have a chance to experience them via means such as a taster session.

While we observed some consistency between parents' and girls' perspectives our findings suggest that recruitment and retention efforts may benefit from targeting the factors each group deem to be most important. For example to garner the support of parents it appears that information about the health and skill development benefits of dance to their daughter and the professionalism of the dance teacher are important. These factors may not resonate so strongly with the girls whose recruitment campaign may be better targeted at the content of the sessions such as music and dance genre, opportunities to socialise, having fun and ownership of dance sessions.

### Strengths and limitations

The major strength of this study is the collection of in-depth information from adolescent girls and their parents about the factors that might influence recruitment and retention in an extra-curricular dance programme. Although we have focussed on dance as the activity of choice we have yielded key information that is likely to be of direct use to researchers or activity providers who may be designing physical activity programmes for adolescent girls. The data were obtained from children living in different economic areas within a single UK city which limits our ability to generalise our findings to other countries or contexts. The sample was also not sufficiently large to specifically test for differences between the economic groups. It is important to note that although the recruitment rate of around a quarter is somewhat low, it is comparable to previous qualitative work with children [41,42] and the focus group transcripts clearly indicate that there was a range of dance and activity interests among the girls. Equally, the sample size for the parental interviews may appear small and only included 2 fathers, but there was clear evidence of saturation where no new information was obtained in both the student focus groups and parental interviews. We are therefore confident that the data presented here are an accurate representation of the views of children and parents in the four schools that participated in the study.

## Conclusions

The data presented in this paper have shown that adolescent girls' recruitment and retention in after-school dance programme could be enhanced by highlighting the enjoyment and socialisation opportunities of the activity. Taster sessions that provide participants with the opportunity to sample the activity and word of mouth campaigns that are facilitated by peer support and encouragement are likely to also encourage girls who are not currently active or do not dance into the activities. Once recruited into the programmes strategies that enable participants to provide input into the sessions and set goals are likely to facilitate retention. Each of these aspects is also likely to be relevant to extra-curricular physical activity interventions that utilise a different form of physical activity. All of the factors identified by the girls and their parents as ways to increase recruitment and retention are consistent with the central tenets of well-established theories of individual behaviour change such as SDT and SCT. The data reported here therefore imply that recruitment and retention campaigns that are targeted to address key mediators might be more effective than non-tailored recruitment and retention efforts and research that examines these issues in relation to extra-curricular physical activity would be helpful.

## Competing interests

The authors declare that they have no competing interests.

## Authors' contributions

The study was conceived by RJ, AC, AH and JP. Data were collected by LD, JM and SJS. Analysis was performed by LD, JM and SJS. RJ drafted the first version of the manuscript with additional sections provided by all authors. All authors provided critical edits and revisions to the paper and have reviewed and approved the final version of the paper.
